# Hotspots of species loss do not vary across future climate scenarios in a drought‐prone river basin

**DOI:** 10.1002/ece3.6597

**Published:** 2020-08-08

**Authors:** Kenneth C. Gill, Rachel E. Fovargue, Thomas M. Neeson

**Affiliations:** ^1^ Department of Geography and Environmental Sustainability University of Oklahoma Norman OK USA

**Keywords:** climate, commonness, conservation, freshwater, prioritization, rarity, uncertainty

## Abstract

Climate change is expected to alter the distributions of species around the world, but estimates of species’ outcomes vary widely among competing climate scenarios. Where should conservation resources be directed to maximize expected conservation benefits given future climate uncertainty? Here, we explore this question by quantifying variation in fish species’ distributions across future climate scenarios in the Red River basin south‐central United States. We modeled historical and future stream fish distributions using a suite of environmental covariates derived from high‐resolution hydrologic and climatic modeling of the basin. We quantified variation in outcomes for individual species across climate scenarios and across space, and identified hotspots of species loss by summing changes in probability of occurrence across species. Under all climate scenarios, we find that the distribution of most fish species in the Red River Basin will contract by 2050. However, the variability across climate scenarios was more than 10 times higher for some species than for others. Despite this uncertainty in outcomes for individual species, hotspots of species loss tended to occur in the same portions of the basin across all climate scenarios. We also find that the most common species are projected to experience the greatest range contractions, underscoring the need for directing conservation resources toward both common and rare species. Our results suggest that while it may be difficult to predict which species will be most impacted by climate change, it may nevertheless be possible to identify spatial priorities for climate mitigation actions that are robust to future climate uncertainty. These findings are likely to be generalizable to other ecosystems around the world where future climate conditions follow prevailing historical patterns of key environmental covariates.

## Graphical abstract

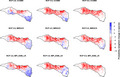

## 1 INTRODUCTION

Climate change is widely expected to alter the distributions and abundances of species around the world (Chen, Hill, Ohlemüller, Roy, & Thomas, 2011; Thomas et al., 2004; Thuiller, 2004), and there is growing interest in conservation strategies that buffer species against potential climate impacts. These strategies include a range of direct and indirect mitigation efforts, including the identification of climate refugia (Ashcroft, 2010; Keppel et al., 2012), the establishment or maintenance of migration corridors (Neeson et al., 2015; Nuñez et al., 2013; Sawyer, Kauffman, Nielson, & Horne, 2009), efforts to predict which ecosystems may be most at risk of being impacted by climate‐related species invasions or pathogens (Scholze, Knorr, Arnell, & Prentice, 2006), and the identification of consistent patterns of range distribution shifts under climatic uncertainty (Lawler & Michalak, 2018; Morin & Thuiller, 2009; Wiens, Stralberg, Jongsomjit, Howell, & Snyder, 2009). At a fundamental level, all of these conservation strategies depend on an ability to predict how species’ distributions and abundances may shift under future climate scenarios.

To estimate how climate change may alter the distributions and abundances of species, researchers often use future climate projections to drive mathematical models of species distributions and abundances (Araújo & New, 2007; Fitzpatrick & Hargrove, 2009; Pearson & Dawson, 2003). Uncertainty and variability in these projections stem from several sources. Nearly all climate projections are generated using a general circulation model (GCM) parameterized with a particular representative concentration pathway (RCP), that is, a future greenhouse gas scenario. A wide range of GCMs is available (Hayhoe et al., 2017); while they differ in their projections and biases, there is widespread agreement that multiple GCMs may be equally valid or appropriate for a given geographic region. For regional analyses, gridded GCM predictions are often mathematically downscaled to create higher resolution gridded projections of temperature, precipitation and runoff. As with GCMs, there is a wide range of mathematical downscaling techniques in use, and the strengths, weaknesses, and biases differ among downscaling methods (Wilby & Wigley, 1997; Wilby et al., 1998). Thus, the combination of multiple GCMs, RCPs, and downscaling technique creates a wide range of future climate projections for any region. Species distribution models (SDMs) add further variability and uncertainty into projections of species’ outcomes. SDMs use a suite of environmental covariates from a given climate scenario to produce a probability of occurrence map for a species (Austin & Van Niel, 2011; Elith et al., 2006). As with GCMs, there are a number of different SDM approaches, and they too differ in their projections and biases (Beaumont, Hughes, & Pitman, 2008; Elith, Kearney, & Phillips, 2010). Thus, projections of species’ distributions may vary widely across future climate scenarios, with the range in outcomes stemming from a combination of exogenous driving variables (e.g., RCPs) and choice of modeling technique and variables (i.e., choice of GCM, downscaling method, and SDM).

Given the considerable uncertainty in projected species’ outcomes across climate scenarios, conservation practitioners are divided as to how to best plan for potential future climate impacts. Indeed, some researchers advocate forecast‐free approaches to conservation planning, arguing that climate projections are best ignored given the uncertainty associated with them (Game, Lipsett‐Moore, Saxon, Peterson, & Sheppard, 2011; Groves et al., 2012). However, an alternative approach is to focus on what conclusions might be drawn despite this uncertainty (Lawler & Michalak, 2018). At the species level, conservation practitioners might focus on identifying the species most at risk (Dirnböck, Essl, & Rabitsch, 2011; Lawler, White, Neilson, & Blaustein, 2006; Ohlemüller et al., 2008), or quantifying the mean and variability of range width shifts for each species across climate scenarios (Cheaib et al., 2012; Morin & Thuiller, 2009; Thuiller, 2004). In a spatial planning framework, researchers might focus on identifying specific locations that are likely to be hotspots of species loss across a wide range of scenarios (Beaumont et al., 2011; Thomas et al., 2004). In this application, a key challenge is to identify specific locations that have consistent biological outcomes (e.g., species loss) across a range of future climate scenarios.

**Figure 1:**
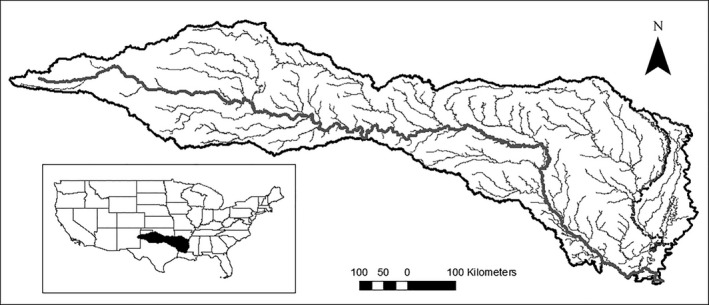
Main stem and major tributaries of the Red River basin. Inset gives the location of the river basin with the continental United States

Arid and semi‐arid river basins are a good model system for exploring these problems, because the key factors that control stream fish species distributions (water availability and temperature) often vary widely among future climate scenarios (Zamani Sabzi, Moreno, et al., 2019; Zamani Sabzi, Rezapour, Fovargue, Moreno, & Neeson, 2019; Zhang, Xu, Tao, Jiang, & Chen, 2010). We focus on the Red River of the South, a large drought‐prone river basin in the southern Great Plains of the United States (Figure 1). Like many Great Plains rivers, the Red River exhibits a dramatic east–west gradient in water availability: The western portion of the basin is very arid, while the eastern portion receives much more precipitation (Matthews, Vaughn, Gido, & Marsh‐Matthews, 2005).

The impacts of climate change are likely to differ among fish species in the Red River basin, with consequences for conservation and management. Stream fishes in the basin are impacted by a wide range of stressors including declining water availability, habitat fragmentation, water quality, and potential climate impacts (Annis, Diamond, Garringer, Hanberry, & Morey, 2012; Labay & Hendrickson, 2014; Perkin & Gido, 2011), but the relative importance of each stressor varies among species. Thus, the potential effects of climate change on species’ distributions likely vary widely among species. We hypothesize that species whose distributions are constrained by nonclimate factors (e.g., habitat fragmentation) may exhibit smaller distributional changes due to climate change than those species whose distributions are constrained by climate‐driven stressors (e.g., water availability or stream temperatures). We also hypothesize that spatial patterns of climate change are independent of spatial patterns of species’ commonness and rarity across the basin. As a result, we expect that common and rare species will on average experience similar proportional changes to their distributions due to climate change, with the result that absolute change will be higher for common (i.e., widespread) species than for narrowly distributed ones. Understanding how absolute and proportional changes in distribution might differ between common versus rare species is of growing interest given recent calls for directing conservation resources toward both common and rare species (Gaston, 2010).

Here, we investigate two questions related to conservation planning for climate mitigation in the Red River: which fish species are most likely to be impacted by climate change, and where are the hotspots of highest species loss across future climate scenarios? For both questions, our aim was to quantify species’ outcomes both within and across climate projections, with a focus on quantifying uncertainty and variability in species’ outcomes. To answer these questions, we first fit SDMs for 31 riverine fish species using historical environmental covariates for the Red River basin. We then used these fitted species distribution models to project the distribution of each species under a range of future climate conditions derived from recent high‐resolution climatic and hydrologic modeling of the basin. Using these projected future distributions, we summarized inter‐ and intraspecies variability in future stream fish distributions across climate scenarios and quantified hotspots of species loss across future climate scenarios.

## 2 METHODS

There are over 150 species of fish in the Red River Basin (Annis et al., 2012). For our analysis, we selected a subset of 31 of these species that collectively span a range of spawning modalities, distributional extent, conservation status, and recreational value (Table 1). These 31 species were chosen to be generally representative of the management priorities of regional conservation practitioners and fisheries agencies based on consultation with K. Kuklinski and T. Sparks, OK Dept. of Wildlife Conservation; and with B. Matthews and E. Marsh‐Matthews, U. Oklahoma.

**Table 1:** The 31 stream fish species used in this analysis. For each species, column headings give common and scientific name; spawning mode (as given by Hoagstrom, Brooks & Davenport, 2011; Perkin & Gido, 2011; Hoagstrom & Turner, 2015); and conservation status according to the state‐level Species of Greatest Conservation Need (SGCN) lists (any tier), IUCN Red List and NatureServe conservation status assessments

Common name	Scientific name	Spawning mode	SGCN Listing	IUCN Red List	NatureServe
Plains Minnow	*Hybognathus placitus*	Pelagic‐broadcast	Species of Concern	Least Concern	Apparently Secure
Prairie chub	*Macrhybopsis australis*	Pelagic‐broadcast	Not listed	Vulnerable	Vulnerable
Red River Shiner	*Notropis bairdi*	Pelagic‐broadcast	Species of Concern	Least Concern	Apparently Secure
Shoal Chub	*Macrhybopsis hyostoma*	Pelagic‐broadcast	Not listed	Least Concern	Secure
Silver Chub	*Macrhybopsis storeriana*	Pelagic‐broadcast	Not listed	Least Concern	Secure
Bigeye Shiner	*Notropis boops*	Riverine	Not listed	Least Concern	Secure
Blackspot Shiner	*Notropis atrocaudalis*	Riverine	Species of Concern	Least Concern	Apparently Secure
Bluehead Shiner	*Pteronotropis hubbsi*	Riverine	Species of Concern	Near Threatened	Vulnerable
Chub Shiner	*Notropis potteri*	Riverine	Species of Concern	Least Concern	Apparently Secure
Emerald Shiner	*Notropis atherinoides*	Riverine	Not listed	Least Concern	Secure
Kiamichi Shiner	*Notropis ortenburgeri*	Riverine	Not listed	N/A	Vulnerable
Ouachita Shiner	*Lythrurus snelsoni*	Riverine	Species of Concern	Least Concern	Vulnerable
Peppered Shiner	*Notropis perpallidus*	Riverine	Species of Concern	Vulnerable	Vulnerable
Plains Killifish	*Fundulus zebrinus*	Riverine	Not listed	Least Concern	Secure
Red Shiner	*Cyprinella lutrensis*	Riverine	Not listed	Least Concern	Secure
Rocky Shiner	*Notropis suttkusi*	Riverine	Species of Concern	*N*/A	Vulnerable
Sand Shiner	*Notropis stramineus*	Riverine	Not listed	Least Concern	Secure
Suckermouth Minnow	*Phenacobius mirabilis*	Riverine	Not listed	Least Concern	Secure
Channel Darter	*Percina copelandi*	Egg burrier or attacher	Not listed	Least Concern	Apparently Secure
Creole Darter	*Etheostoma collettei*	Egg burrier or attacher	Species of Concern	Least Concern	Apparently Secure
Leopard Darter	*Percina pantherina*	Egg burrier or attacher	Species of Concern	Endangered	Imperiled
Orangebelly Darter	*Etheostoma radiosum*	Egg burrier or attacher	Species of Concern	Least Concern	Secure
Red River Pupfish	*Cyprinodon rubrofluviatilis*	Egg burrier or attacher	Species of Concern	Least Concern	Secure
Blue Catfish	*Ictalurus furcatus*	Nesting	Not listed	Least Concern	Secure
Black Bullhead	*Ameiurus melas*	Nesting	Not listed	Least Concern	Secure
Green Sunfish	*Lepomis cyanellus*	Nesting	Not listed	Least Concern	Secure
Largemouth Bass	*Micropterus salmoides*	Nesting	Not listed	Least Concern	Secure
Smallmouth Bass	*Micropterus dolomieu*	Nesting	Not listed	Least Concern	Secure
Spotted Bass	*Micropterus punctulatus*	Nesting	Not listed	Least Concern	Secure
Striped Bass	*Morone saxatilis*	Nesting	Not listed	Least Concern	Secure
Western Mosquitofish	*Gambusia affinis*	Livebearer	Not listed	Least Concern	Secure

For each of the 31 species, we gathered historical occurrence records from the Global Biodiversity Information Facility (GBIF, www.gbif.org). GBIF serves as one of the most extensive biogeographical resources in the world (Beck, Böller, Erhardt, & Schwanghart, 2014) and collates species occurrence records from peer‐reviewed research articles and agency and museum collections. We automated the collection of species occurrence records from GBIF using the R package “dismo” (Hijmans, Phillips, Leathwick, Elith, & Maintainer, 2017), which includes automated removal of duplicate records. Because our intent was to focus on stream fish distributions, we also excluded occurrence records from reservoirs. The temporal range of occurrence records spanned the years 1919–2010; however, only about 1% of occurrence records predate 1960. Verifying that each species’ occurrence points are statistically random (ROR) is a necessary step in production of unbiased MaxEnt models (Merow, Smith, & Silander, 2013). Our initial analysis of these historical occurrence data revealed that they have a statistically random relative occurrence rate (ROR), which ranges from 30 to 1,576 historical records per species.

### 2.1 Overview of modeling approach

Our modeling approach proceeded in three steps. First, we used historical occurrence records and a suite of spatial covariates describing the recent historical environment to fit a species distribution model (SDM) for each species. These fitted SDMs describe the historical probability of occurrence of each species across the Red River basin. Second, we projected the future distribution of each species under a range of potential climate scenarios by coupling our fitted species distribution models with projected values of climatic and hydrologic variables under future climate scenarios. Third, we summarized inter‐ and intraspecies variability in future stream fish distributions across these climate scenarios.

We selected covariates for our SDM analysis by choosing environmental factors within the Red River Basin which are known to drive the distribution of stream fish species and are commonly used for modeling stream fish distributions (Annis et al., 2012; Bond, Thomson, Reich, & Stein, 2011; Labay & Hendrickson, 2014). Our analysis includes two classes of covariates (Table 2). One set of covariates is temporally dynamic and includes those that vary among historical and future climate scenarios. The other set of covariates is temporally static; these covariates were assumed to be constant across both historical and future climate scenarios.

**Table 2:** Bioclimatic variables used as predictors in species distribution models. Columns give the data sources; and whether the variable is temporally static or dynamic (i.e., differs among climate scenarios)

Covariate name	Source	Covariate type
Annual mean temperature	SC‐CASC	Dynamic
Annual mean rainfall	SC‐CASC	Dynamic
Mean temperature of wettest quarter	SC‐CASC	Dynamic
Mean temperature of driest quarter	SC‐CASC	Dynamic
Mean annual flow	SC‐CASC	Dynamic
Mean flow of wettest quarter	SC‐CASC	Dynamic
Mean flow of driest quarter	SC‐CASC	Dynamic
Strahler stream order	NHD	Static
Anthropogenic barrier density	NABD	Static
Elevation	USGS	Static
Lithology class	USGS	Static
Land cover class	NLCD	Static
Habitat disturbance index	NFHAP	Static

Temporally dynamic covariates were drawn from recent high‐resolution climatic and hydrologic modeling of the basin (Bertrand & McPherson, 2018; McPherson, 2016; Xue et al., 2015). McPherson et al. (2016) used statistical downscaling of global climate model outputs to create estimates of air temperature and precipitation across the basin at a 1/8° raster resolution for both historical time series (the years 1961–2010) and for future climate scenarios (the years 2010–2099). McPherson et al. created these downscaled air temperature and precipitation estimates for a total of nine climate scenarios, which resulted from taking all combinations of three general circulation models (GCMs; CCSM4, MIROC5, and MPI_ESM_LR) and three representative concentration pathways (RCPs; 2.6, 4.5, and 8.5 W/m^2^). These nine scenarios were selected based on air temperature and precipitation biases over the south‐central United States (McPherson et al., 2016) following the recommendations of Sheffield et al. (2013). Air temperature and precipitation estimates for each of these nine climate scenarios were then used to drive a Variable Infiltration Capacity (VIC) hydrologic model to simulate surface runoff, streamflow, and reservoir storages for both historical and future time periods using the same 1/8° resolution raster grid used for climate projections. VIC is a rainfall‐runoff model that uses climate variable inputs (precipitation, temperature), estimates of infiltration and soil moisture, and reservoir storages to estimate evapotranspiration and surface runoff at a daily time step across the basin (Liang, Wood, & Lettenmaier, 1996). Details of the VIC model calibration process for the Red River basin are given by Xue et al. (2015). For each of the nine climate scenarios, we calculated climatic and hydrologic covariates from the VIC model output with a focus on temperature, streamflow, and drought covariates hypothesized to influence stream fish distributions in the region (Table 2; Labay & Hendrickson, 2014). Specifically, we calculated annual mean air temperature, annual mean precipitation, mean temperatures of the driest and wettest quarters, mean annual surface flow, and the mean flows of the wettest and driest quarter for each 1/8° resolution raster grid cell in the basin.

Temporally static covariates were drawn from a range of widely available spatial data sets and spatially averaged over each 1/8° resolution raster grid used for climate and VIC model outputs. We extracted Strahler stream order from the National Hydrography Dataset, elevation from the USGS 30 m DEM, and categorical land cover data from the 2011 National Land Cover Data Set. We included lithology (soil type) to serve as a proxy for conductivity, which is known to be an important driver of fish assemblages in portions of the Red River basin (Pyron & Taylor, 1993). Because stream fragmentation is widely acknowledged to be a key driver of stream fish assemblages (Perkin & Gido, 2011), we included an index of anthropogenic barrier density as a covariate in our SDMs. Specifically, we calculated the density of anthropogenic barriers in each 1/8° raster cell using the National Anthropogenic Barrier Dataset. We also included the disturbance index from the National Fish Habitat Action Plan (Crawford et al., 2016) to account for habitat condition and nonfragmentation anthropogenic disturbances on each stream reach.

### 2.2 Species distribution models

Commonly used methods for fitting species distribution models include generalized linear models (GLMs), generalized additive models (GAMs), boosted regression trees (BRTs), and the MaxEnt maximum entropy model (Elith et al., 2010). We chose to use MaxEnt for our species distribution modeling for two reasons. First, our species data contain only historical occurrence points (not absences), and MaxEnt is the most appropriate choice for modeling presence‐only data (Elith & Graham, 2009). Second, a key objective of our study was to use the fitted species distribution models to project species’ distributions under future climate scenarios. MaxEnt is the preferred model for extrapolating species’ distributions to new environments because it is “clamped,” that is, it extrapolates in a horizontal line from the most extreme environmental values in the training data set (Elith & Graham, 2009; Elith et al., 2011). Furthermore, MaxEnt models are commonly used for modeling stream fish distributions (Annis et al., 2012; Bond et al., 2011; Labay & Hendrickson, 2014).

For each of the 31 species in our data set, we fit a MaxEnt model using all temporally static covariates and historical period temporally dynamic covariates (Table 2). Our spatial unit of analysis for the MaxEnt modeling was the 1/8° resolution raster grid cells used for climate and hydrological modeling. We aggregated point records of historical species’ occurrences to these grid cells by assuming that a species was present in a grid cell if that grid cell contained at least one historical occurrence record.

A previous analysis isolating the Bluehead Shiner (*Pteronotropis hubbsi*) in the Red River Basin found that optimizing the regularization multiplier between 1.5× and 2.0× was necessary to prevent over‐prediction while staying under the target training omission rate of 30% (Hernandez, 2015). Thus, we optimized the regularization multiplier within MaxEnt to give more predictive power to the covariates that have the most influence and to penalize the variables which do not influence the model outputs. Additionally, we used a jackknife approach in our MaxEnt model to quantitatively identify the most influential covariates. By increasing the regularization multiplier, our model generally produced a broader range of projected occurrence probabilities and is better fitted with respect to model area under the curve (AUC) values (Hernandez, 2015; Radosavljevic & Anderson, 2014). We assessed model fit by focusing on AUC values for the fitted models. Values between 0.7 and 0.9 are considered “usable” while values above 0.9 are considered excellent (Swets, 1988).

To project fish distributions under each of the nine future climate scenarios, we used projected future values of all environmental covariates (Table 2) as drivers to the fitted MaxEnt models. Thus, our projections of fish distributions represent an extrapolation of the fitted MaxEnt models (i.e., the models parameterized with historical covariates) into each of the nine future climate scenarios. We summarized projected species’ distributions within and across climate scenarios in several ways. First, we calculated the projected change in the distribution of each species in each climate scenario by comparing projected to historical distributional extents. For this calculation, we used a projected probability of occurrence of 0.5 as a threshold for determining whether a raster cell should be included in each species’ range. While this choice of threshold may not maximize prediction accuracy for every species (Freeman & Moisen, 2008), our priority was to use an identical threshold for all species to enable us to compare habitat gains and losses across species in an even‐handed way. Thus, we chose to use 0.5 as an occurrence threshold because it is a traditional default choice (Freeman & Moisen, 2008). Second, we estimated hotspots of species loss or gain in each climate scenario by summing the change in probability of occurrence across all species for each raster cell. The resulting output is a raster map for each climate scenario that highlights locations of species loss or gain. Finally, we created a single map to summarize outcomes across climate scenarios by summing all changes in probability of occurrence for each raster cell across all species and across all climate scenarios.

## 3 RESULTS

Environmental covariates related to air temperature, flow, lithology, and elevation had the greatest influence on species’ distributions (Table 3), as measured by percent contribution in the fitted MaxEnt models (Phillips, Anderson, & Schapire, 2006). Among covariates that change across future climate scenarios, temperature and streamflow during the driest quarter of the year (i.e., summer) were particularly important: the mean temperature of the driest quarter of the year had the second greatest average influence across all species, and the mean streamflow during the driest quarter had the third greatest influence overall. The two most important static covariates (i.e., those did not vary among climate scenarios) were lithology (highest influence overall) and elevation (4th overall). In terms of overall model fit, we found that historical distributions of stream fishes were well explained by MaxEnt models; the majority of models (22 of 31 species) had an area under the curve (AUC) value of 0.85 or greater, indicating near‐excellent fits.

**Table 3:** Average percent contribution of each covariate within fitted historical MaxEnt models, with the average percent contribution taken across all species

Covariate name	Average percent contribution
Lithology type	29.14
Mean air temperature of driest quarter	21.37
Mean flow of driest quarter	12.00
Elevation	9.46
Mean annual rainfall	8.03
Mean annual air temperature	4.74
Mean air temperature of wettest quarter	4.64
Mean annual flow	2.63
Land cover	2.35
NFHAP disturbance index	1.93
NABD density	1.87
Mean flow of wettest quarter	1.09
Strahler stream order	0.66

**Figure 2:**
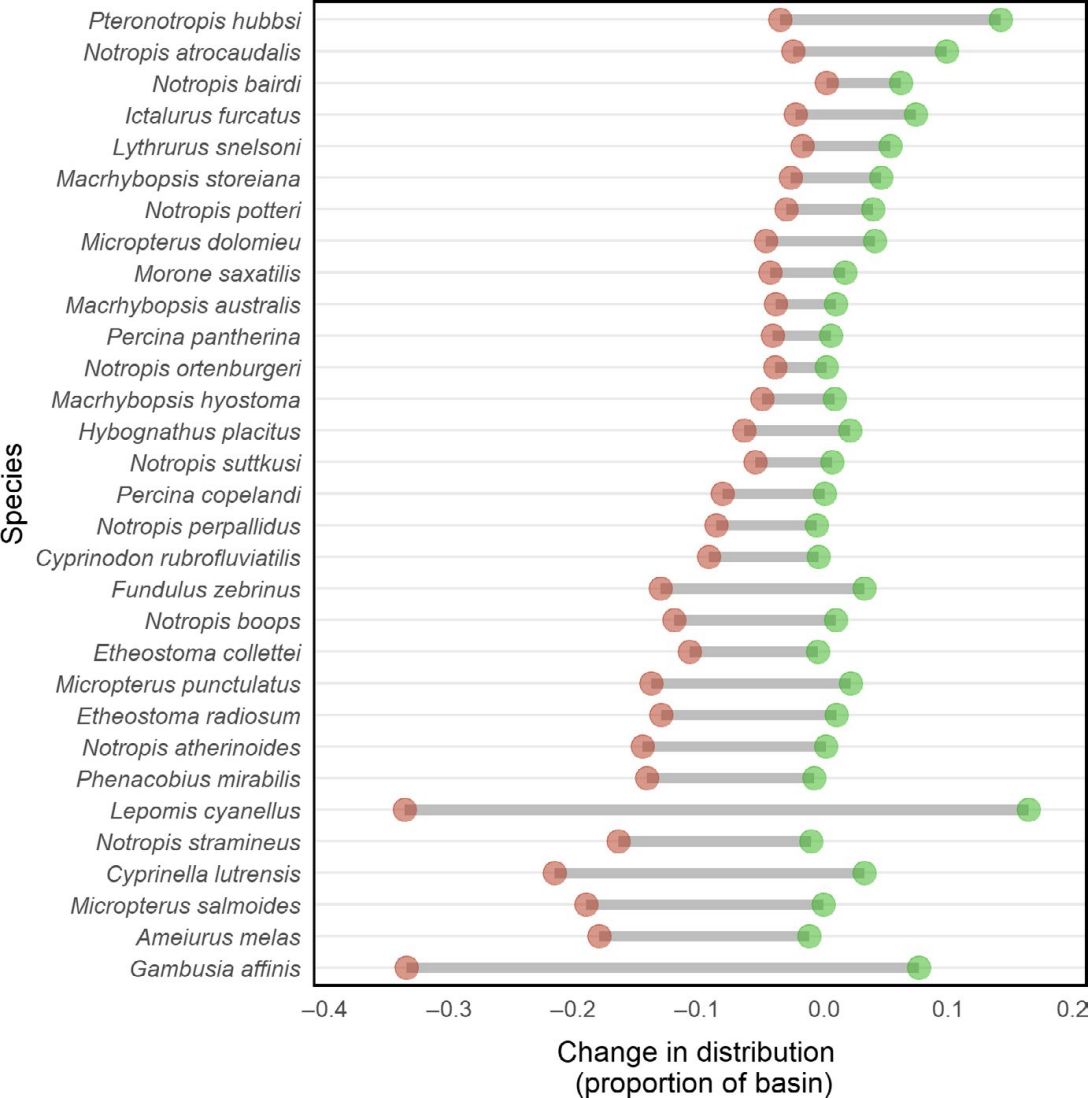
Variability in changes to species’ distributional extents among nine future climate scenarios in the Red River basin, as predicted by MaxEnt. For each species, the horizontal axis gives the difference in the proportion of the basin where that species is projected to occur (based on a threshold probability of occurrence of 0.5) between the historical period and the year 2050. The endpoints of each bar give the minimum and maximum observed range shifts across nine climate scenarios

Species differed markedly in projected changes to their distributional range under future climate scenarios, and also in the variability of these projected outcomes across climate scenarios (Figure 2). For example, the MaxEnt model suggests that the distributional range of *Notropis atrocaudalis* and *P. hubbsi* will increase or remain similar in the future in all nine future climate scenarios. Conversely, MaxEnt suggests that *Ameiurus melas*, *Micropterus salmoides*, and others will be more narrowly distributed in the future. For some species, projected changes to their distribution are similar across all nine climate scenarios. In the case of *Morone saxatilis*, for example, the most optimistic and pessimistic climate scenarios are similar (expanding to an additional 0.52% of the basin versus. disappearing from 1.59% of the basin, respectively). For other species, like *Gambusia affinis* and *Lepomis cyanellus*, changes to their projected distributional extent vary widely across the nine climate scenarios. For *G. affinis*, its distribution is projected to increase to encompass an additional 7.6% of the basin under the most optimistic climate scenario (under GCM MIROC5 and RCP 4.5) in 2050 under MaxEnt. However, the most pessimistic climate scenario is dramatically different and suggests that its future distribution will contract and fail to include 34% of the basin where it historically occurred (under GCM MPI_ESM_LR and RCP 4.5).

**Figure 3:**
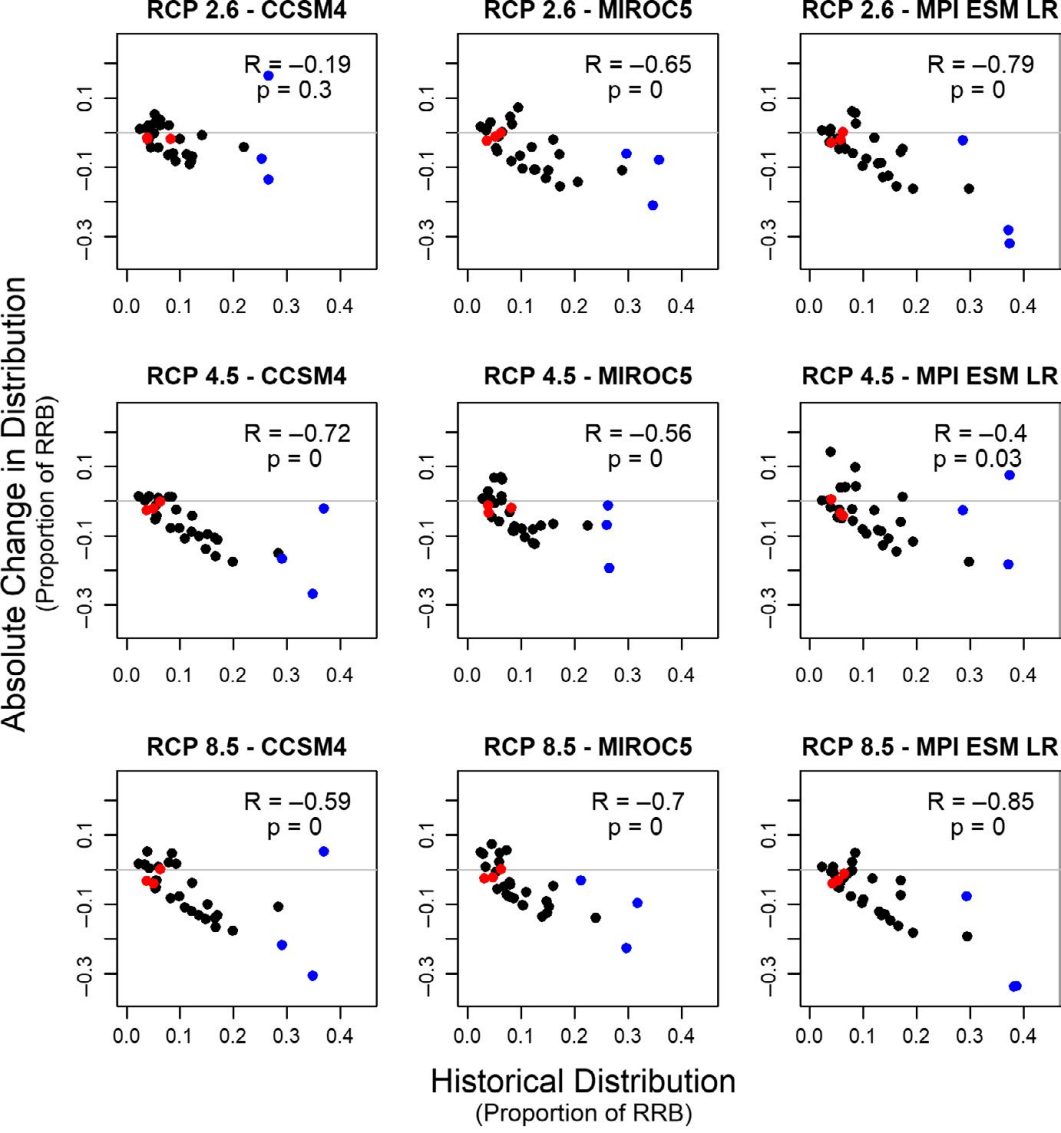
Comparison of historical range extent (as proportion of the Red River basin [RRB]; horizontal axis) versus absolute change in range extent in the year 2050 (as proportion of the Red River basin; vertical axis) for nine climate scenarios, as predicted by MaxEnt. Each point represents one species. Colored points highlight species that were historically common (blue) or rare (red) and are discussed in the main text

**Figure 4:**
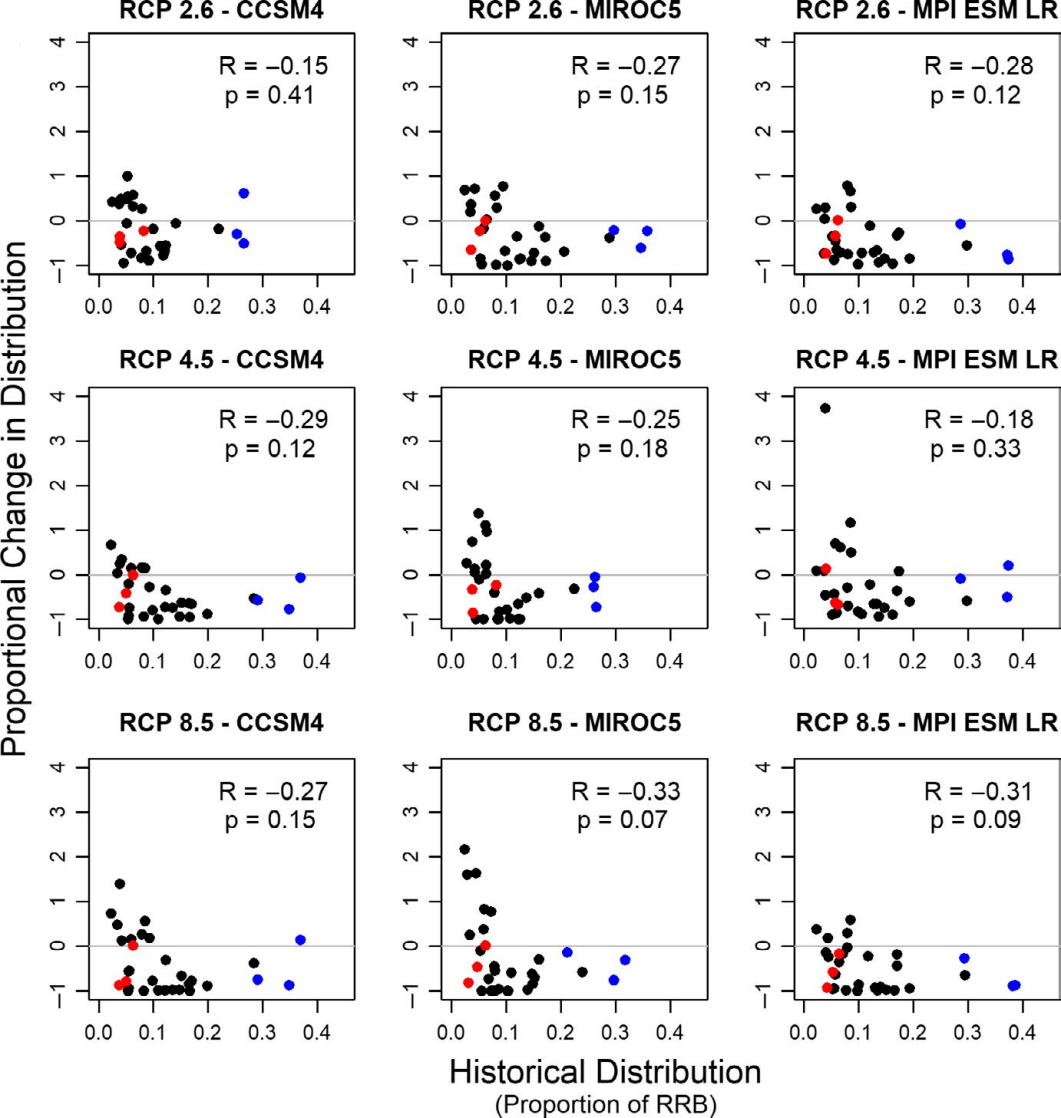
Comparison of historical range extent (as proportion of the Red River basin [RRB]; horizontal axis) versus proportional change in range extent in the year 2050 (compared to historical distribution; vertical axis) for nine climate scenarios, as predicted by MaxEnt. Each point represents one species. Colored points highlight species that were historically common (blue) or rare (red) and are discussed in the main text

We also found that the greatest absolute changes in distribution under future climate scenarios are projected to occur for the most widely distributed species (Figure 3). Across eight of nine climate scenarios, we found a statistically significant negative correlation between historical distributional extent and the absolute change in distributional extent in 2050. For example, *G. affinis*, *L. cyanellus*, and *Cyprinella lutrensis* are all historically widely distributed across the basin, but are projected to occupy a much narrower portion of the basin in all future climate scenarios. Conversely, absolute changes to the distributional range of several species that were historically narrowly distributed (e.g., *Percina pantherina*, *Notropis ortenburgeri*, and *Macrhybopsis australis*) were small because those species were rare to begin with. However, we did not find a significant correlation between historical distributional extent and proportional change in distributional extent in 2050 (Figure 4); thus, we could not reject a null hypothesis that changes to species’ distributional extents were proportional to their historical distributional extent.

We observed significant differences in distributional changes among groups of species. The median outcome across climate scenarios (i.e., the midpoint of outcomes for each species in Figure 2) was significantly better for species of greatest conservation need (mean change in distribution = −1.1% of basin; *n* = 12 species) than for species that were not SGCN listed (mean = −5.2%; *n* = 19; *t* test, *p* < .05). We also found that the median outcome across climate scenarios was significantly better for pelagic‐broadcast spawners (mean change in distribution = −0.3% of basin; *n* = 5 species) than for all other species (mean = −4.2%; *n* = 26; *t* test, *p* < .05). However, we did not find significant differences in outcomes among species when comparing all spawning modes (ANOVA; *p* > .05); here, we compared pelagic‐broadcast spawners (mean change in distribution = −0.3% of basin; *n* = 5 species), riverine spawners (mean = −3.2%; *n* = 13), egg burriers or attachers (mean = −4.5%; *n* = 5 species), and nesting species (mean = −4.7%; *n* = 7 species).

**Figure 5:**
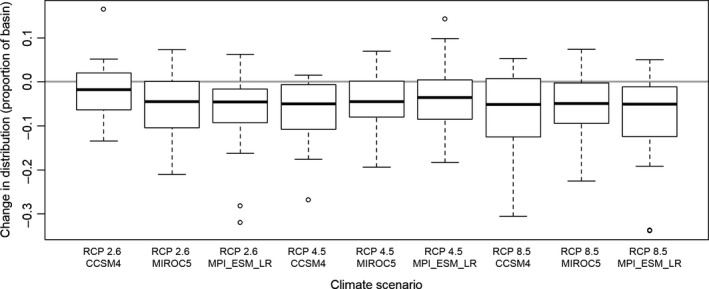
Boxplots summarizing variation among species in projected changes to their distributional extent across nine climate scenarios, as predicted by MaxEnt models. For each species, change in distribution is the difference in the proportion of the Red River basin where that species is projected to occur (based on a threshold probability of occurrence of 0.5) between the historical period and the year 2050

While there were large differences in how individual species fared among climate scenarios (Figure 2), the average change in range width across the entire fish community was similar across climate scenarios (Figure 5). The effects of RCP emission scenario and GCM model choice appeared to be modest. For example, most species experienced the best outcomes (i.e., least amount of range contraction) in the RCP 2.6 and the CCSM4 GCM, but differences were small relative to the other 8 eight climate scenarios. Similarly, the RCP 8.5 scenarios included the highest number of species that were projected to disappear from more than 20% of the basin; however, average outcomes for all three RCP 8.5 scenarios were still similar to the remaining six climate scenarios.

**Figure 6:**
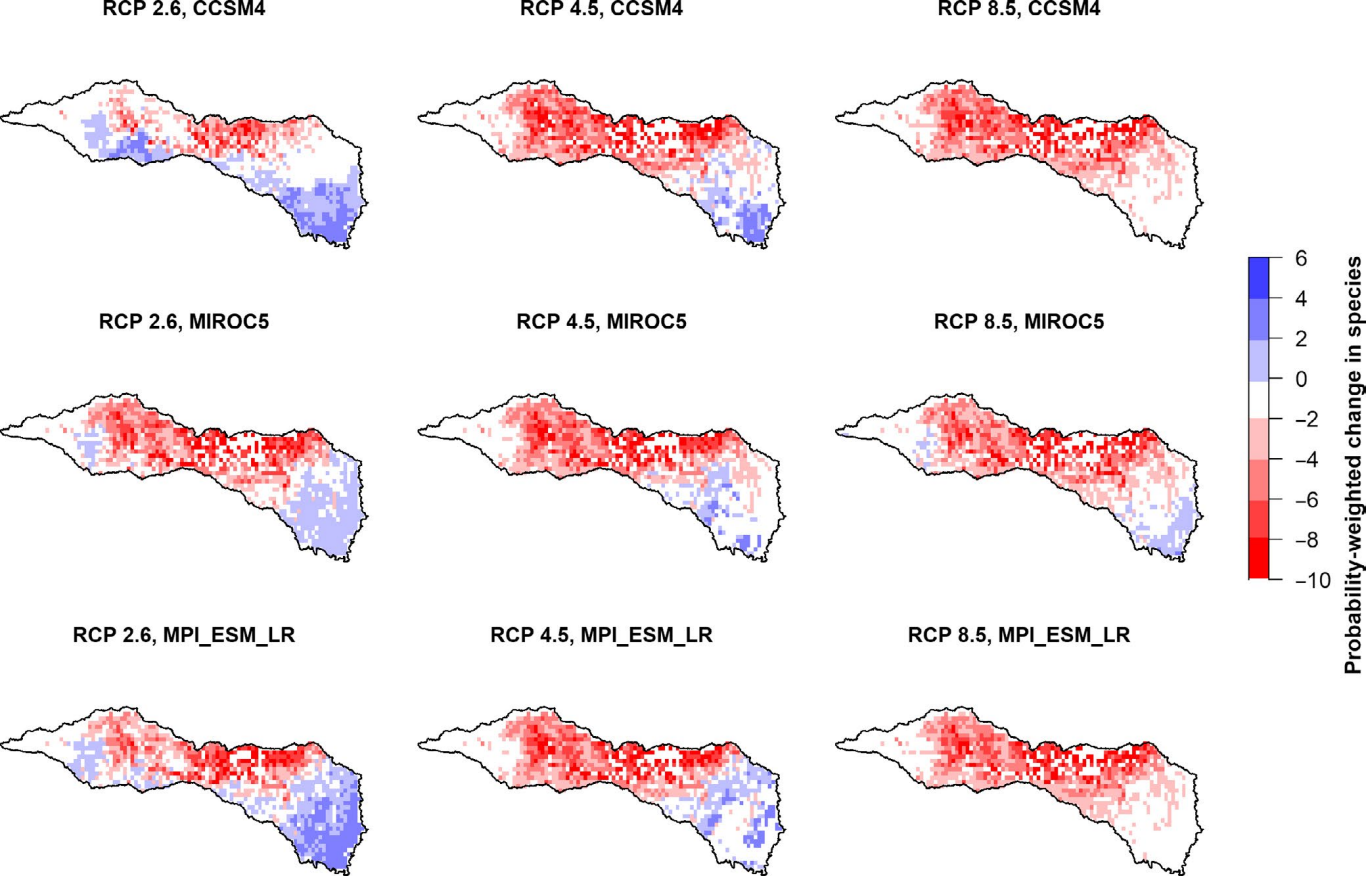
Expected change in the number of species within the Red River basin by the year 2050 across nine future climate scenarios, as projected by MaxEnt. The value for each raster 1/8° cell represents the difference in species’ probability of occurrence between the historical period and the year 2050, summed across all species

**Figure 7:**
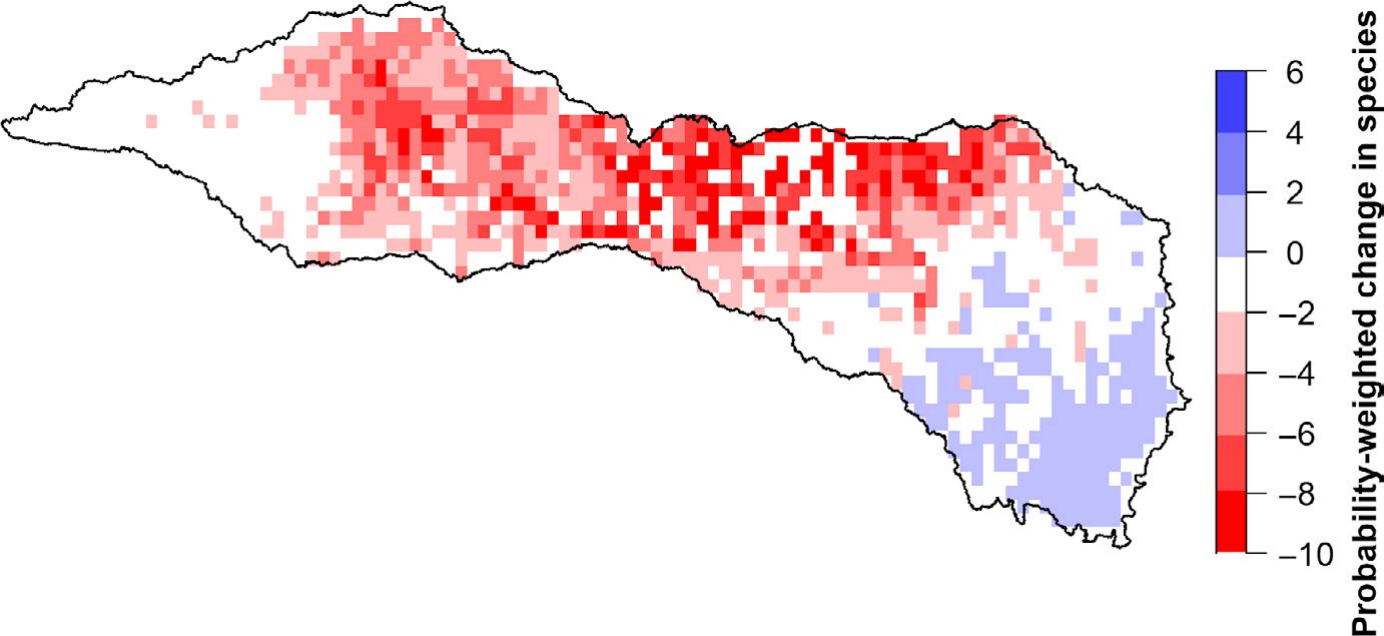
Mean expected change in the number of species within the Red River basin by the year 2050, averaged across nine future climate scenarios, as projected by MaxEnt. The value for each raster 1/8° cell represents the difference in species’ probability of occurrence between the historical period and the year 2050, summed across all species and then averaged across nine climate scenarios (i.e., raster cell values are an average of the nine maps in Figure 6)

Despite high variability in outcomes for individual species across climate scenarios (Figures 2 and 3), we found that the spatial hotspots of greatest species loss tended to occur in the same locations in all nine climate scenarios (Figure 6). Specifically, we found that the change in probability of occurrence summed across all species was highest in the north‐central portion of the RRB across scenarios. Aggregate outcomes in other portions of the basin varied among climate scenarios. In the southeast corner of the RRB, for example, our models predicted no net change in aggregate probability of occurrence under two scenarios (CCSM4 and MPI_ESM_LR GCMs under RCP 8.5) and an increase in aggregate probability of occurrence in the region for the remaining seven climate scenarios. Taking the average of these scenario‐specific maps (i.e., panels in Figure 6) resulted in a single map (Figure 7) that highlights locations in the basin with high average species loss across all climate projections. Spatial trends in this figure mirror those in panels of Figure 6, such that hotspots of species loss are concentrated in the north‐central portion of the basin.

## 4 DISCUSSION

Our analysis of fish species distributions under nine future climate scenarios highlights a wide range of outcomes across species and across scenarios in the Red River. We found that the range extent of most fish species in the Red River Basin will contract over the next few decades across all GCM/RCP scenarios (Figure 2). Species listed as Species of Greatest Conservation Need experienced less habitat loss, on average, than nonlisted species. We also found that pelagic‐broadcast spawners experienced less habitat loss, on average, than species with other spawning habits. Conversely, species varied dramatically in the uncertainty associated with their future distributions, with the range in outcomes across climate scenarios being more than 10 times greater for some species than for others. Our analysis also revealed that the greatest absolute changes in range width are projected to occur for those species which have been the most widespread historically (Figure 3).

Despite this variability in how individual species fared across climate scenarios, we also found that the hotspots of greatest species loss were consistent across climate scenarios (Figure 6). Overall, then, we find that there is high uncertainty regarding outcomes for individual species, but lower uncertainty regarding spatial hotspots where the negative impacts of climate change are likely to be greatest. Furthermore, our aggregate map of changes to species’ probabilities of occurrence (Figure 7) provides a spatial summary of these hotspots of projected species loss in future climate scenarios.

Our finding that the most common (i.e., widespread) species are projected to face the largest absolute changes in distributional extent (Figure 3) also underscores arguments for including common species in conservation and climate mitigation initiatives. Indeed, there is growing awareness that many common species play key roles in maintaining ecosystem structure and function (Geider et al., 2001; Grime, 1998; Winfree, W. Fox, Williams, Reilly, & Cariveau, 2015), and efforts to conserve rare species can easily lead to neglect of common species (Gaston, 2010; Gaston & Fuller, 2008). Furthermore, conservation investments in common species are often more cost‐effective, because their wide distributional range enables conservation practitioners to choose among many candidate projects, some of which will offer high benefit per dollar spent (Gaston, 2010; Neeson et al., 2018). At the same time, investments in rare species are critical: our models also suggest that several regionally imperiled species are likely to be further impacted by climate change. For example, all climate projections suggest some degree of range contraction for the federally endangered Leopard Darter (*P. pantherina*). In other cases, species considered as vulnerable by NatureServe (e.g., the Rocky Shiner, *Notropis suttkusi*), experienced dramatic range contraction across all climate scenarios. When considering conservation actions for imperiled species, conservation practitioners should recognize that future climate projections and SDM outputs entail multiple sources of error, and for some species model fit (as measured by AUC) was relatively lower. Thus, the precautionary principle (Kriebel et al., 2001) might be a basis for directing climate mitigation resources toward imperiled species even if they show few climate impacts in most future scenarios.

In most cases, the relative importance of environmental covariates in our models (Table 3) mirrors empirical work on stream fish ecology in the south‐central United States. For example, the mean air temperature of the driest quarter (i.e., summer) was on average the most important covariate in our models. This finding is in line with a wealth of empirical work on the role of summer stream temperatures in structuring stream fish distributions (Eaton & Scheller, 1996; Matthews & Zimmerman, 1990; Ostrand & Wilde, 2001). Similarly, mean flow in summer, our third most important covariate, is also widely acknowledged to be an important control on stream fish distributions (Dodds, Gido, Whiles, Fritz, & Matthews, 2004; Falke, Bestgen, & Fausch, 2010; Perkin et al., 2015). Important static landscape covariates (e.g., lithology and elevation) capture the roles of salinity (Higgins & Wilde, 2005; Ostrand & Wilde, 2001) and local topography (e.g., the extent of the central interior highlands; Mayden, 1985). However, we did not find that the density of anthropogenic barriers (i.e., habitat fragmentation) was an important covariate, despite recent empirical evidence that anthropogenic barriers have a strong influence on regional fish communities (Perkin & Gido, 2011; Perkin et al., 2015; Sleight & Neeson, 2018). Alternative measures of fragmentation (e.g., the dendritic connectivity index; Cote, Kehler, Bourne, & Wiersma, 2009) may have had different influence on the SDM models. Our historical occurrence data spanned a range of decades and included fish occurrence records both before and after construction of many of the reservoirs in the basin; thus, this temporal mismatch may have made it difficult for our SDMs to correctly identify the role of habitat fragmentation in structuring fish communities (Milt et al., 2018). Finally, we recognize that our approach to estimating species richness at a site by summing independent SDMs tends to over‐estimate species richness (Calabrese, Certain, Kraan, & Dormann, 2014) because it does not consider upper limits on the number of species that may occur at a single position.

Our analysis of projected fish species distributions under future climate scenarios highlights opportunities for conservation practitioners and decision makers to make pro‐active investments in fish conservation to mitigate potential climate impacts. Given the importance of stream temperature and flow, conservation practitioners might use our SDM projections to identify specific locations where species’ distributions are likely to contract due to unfavorable stream temperature and flow. In these locations, practitioners may consider strategies to mitigate climate impacts by ensuring adequate instream flows (Arthington, Bunn, Poff, & Naiman, 2006; Poff et al., 2010) and by ensuring that reservoir releases maintain adequate stream temperatures and flows where possible (Guo, Zamanisabzi, Neeson, Allen, & Mistree, 2019; Poff & Zimmerman, 2010; Zamani Sabzi, Moreno, et al., 2019; Zamani Sabzi, Rezapour, et al., 2019). In locations where multiple concurrent stressors are projected to drive species losses, conservation actors may need to evaluate multiple types of conservation actions (Fitzpatrick & Neeson, 2018; Neeson, Smith, Allan, & McIntyre, 2016; Radeloff et al., 2015) and coordinate investments in stream restoration across the basin (Kark, Levin, Grantham, & Possingham, 2009; Milt et al., 2017; Neeson et al., 2015). In some of these locations, mitigation of nonclimate stressors may not pay dividends because future climate conditions will be beyond the tolerance of resident species or otherwise infeasible (Popejoy, Randklev, Neeson, & Vaughn, 2018). In this case, SDM projections might be used to identify locations for facilitating dispersal or passive migration management to locations with more favorable future climate conditions (Guisan et al., 2013).

Climate change is projected to impact species in ecosystems around the world (Chen et al., 2011; Thomas et al., 2004; Thuiller, 2004), but the projected impacts to individual species vary widely among future scenarios. Our work demonstrates how conservation practitioners might seek to both identify those species at greatest risk of climate change (Dirnböck et al., 2011; Morin & Thuiller, 2009; Ohlemüller et al., 2008; Thuiller, 2004) and also work to identify locations that appear to be hotspots of species loss across a wide range of scenarios (Beaumont et al., 2011; Thomas et al., 2004). Conversely, locations that remain within the range of many species across a wide range of future climate scenarios may be good candidate locations for the establishment of climate refugia (Ashcroft, 2010; Keppel et al., 2012). These multiple perspectives underscore how conservation actors might work to pro‐actively mitigate the potential impacts of climate change despite multiple sources of uncertainty in both climate projections and species distribution models (Lawler & Michalak, 2018).

## CONFLICT OF INTEREST

The authors declare that they have no competing interests.

## AUTHOR CONTRIBUTIONS

**Kenneth C Gill:** Conceptualization (equal); Data curation (lead); Formal analysis (lead); Methodology (equal); Validation (equal); Visualization (equal); Writing‐original draft (lead); Writing‐review & editing (equal). **Rachel E Fovargue:** Conceptualization (equal); Formal analysis (equal); Methodology (equal); Visualization (equal); Writing‐review & editing (equal). **Thomas Neeson:** Conceptualization (equal); Formal analysis (equal); Funding acquisition (lead); Methodology (equal); Supervision (lead); Writing‐review & editing (equal).

## Data Availability

Data used for this study are available for download from Dryad at https://doi.org/10.5061/dryad.1g1jwstsn.
